# Benchmarking System Monitoring on Quality Improvement in Percutaneous Coronary Intervention: A Nationwide Registry in Japan

**DOI:** 10.1016/j.jacasi.2023.12.003

**Published:** 2024-02-20

**Authors:** Yuichi Saito, Taku Inohara, Shun Kohsaka, Hideki Wada, Hiraku Kumamaru, Kyohei Yamaji, Hideki Ishii, Tetsuya Amano, Hiroaki Miyata, Yoshio Kobayashi, Ken Kozuma, J-PCI Registry Investigators

**Affiliations:** a Department of Cardiovascular Medicine, Chiba University Graduate School of Medicine, Chiba, Japan; b Department of Cardiology, Keio University School of Medicine, Tokyo, Japan; c Department of Cardiovascular Medicine, Juntendo University Shizuoka Hospital, Izunokuni, Japan; d Department of Healthcare Quality Assessment, Graduate School of Medicine, University of Tokyo, Tokyo, Japan; e Department of Cardiology, Kyoto University, Kyoto, Japan; f Department of Cardiovascular Medicine, Gunma University Graduate School of Medicine, Gunma, Japan; g Department of Cardiology, Aichi Medical University, Nagakute, Japan; h Department of Cardiology, Teikyo University Hospital, Tokyo, Japan

**Keywords:** benchmarking, feedback, percutaneous coronary intervention, quality indicator

## Abstract

**Background:**

Quality indicators (QIs) have been developed to improve and standardize care quality in percutaneous coronary intervention (PCI). In Japan, consecutive PCI procedures are registered in a nationwide database (the Japanese Percutaneous Coronary Intervention registry), which introduces a benchmarking system for comparing individual institutional performance against the national average.

**Objectives:**

The aim of this study was to assess the impact of the benchmarking system implementation on QI improvement at the hospital level.

**Methods:**

A total of 734,264 PCIs were conducted at 1,194 institutions between January 2019 and December 2021. In January 2018, a web-based benchmarking system encompassing 7 QIs for PCI at the institutional level, including door-to-balloon time and rate of transradial intervention, was introduced. The process by which institutions tracked their QIs was centrally monitored.

**Results:**

During the 3-year study period, the benchmarking system was reviewed at least once at 742 institutions (62.1%) (median 4 times; Q1-Q3: 2-7 times). The institutions that reviewed their records had higher PCI volumes. Among these institutions, although door-to-balloon time was not directly associated, the proportion of transradial intervention increased by 2.3% in the system review group during the initial year compared with 0.7% in their counterparts. However, in the subsequent year, the association between system reviews and QI improvement was attenuated.

**Conclusions:**

The implementation of a benchmarking system, reviewed by participating institutions in Japan, was partially associated with improved QIs during the first year; however, this improvement was attenuated in the subsequent year, highlighting the need for further efforts to develop effective and sustainable interventions to enhance care quality in PCI.

Percutaneous coronary intervention (PCI) has emerged as a commonly performed procedure in at-risk patients with acute coronary syndromes (ACS) and stable ischemic heart disease. Nevertheless, significant variations in the quality of care have been observed among physicians, hospitals, and regions.^1^, ^2^, ^3^, ^4^, ^5^, ^6^ To achieve clinical improvements and standardize the quality of care, implementing benchmarking for quality indicators (QIs) within the health care system is effective.^7^ QI initiatives have been proposed and implemented in various areas of medicine to promote the delivery of evidence-based care. The use of QI has garnered the interest of multiple stakeholders, including academic societies, payers, and the public.^2^^,^^8^^,^^9^

Various QIs have been used in the context of PCI, including door-to-balloon (D2B) time in ST-segment elevation myocardial infarction (STEMI) and the implementation of transradial intervention (TRI).^9^^,^^10^ Although adherence to such QIs in these settings has been linked to improved outcomes according to various benchmarking methods,^3^^,^^11^, ^12^, ^13^, ^14^ the optimal approach for providing feedback to enhance compliance with QIs remains uncertain.^15^^,^^16^ These interventions may be one time, regular, real time, interactive, or multifaceted (eg, checklists and educational materials). However, the effectiveness of each approach has yet to be established.^16^, ^17^, ^18^

The Japanese Association of Cardiovascular Intervention and Therapeutics (CVIT), a professional society responsible for certifying both interventionalists and institutions in Japan, recently introduced a benchmarking system that incorporates registered data for consecutive PCI procedures from each site, enabling a comparison of individual institutional performance against the national average. The participating institutions were encouraged to assess their performance biannually upon renewal of their institutional recertification. The aim of this study was to investigate the impact of implementing a feedback system on QI improvement at the hospital level following the introduction of the QI benchmarking mechanism.

## Methods

### Data source

The Japanese Percutaneous Coronary Intervention (J-PCI) registry is an ongoing, prospective, nationwide registry endorsed by CVIT, in which clinical variables and outcome data from patients undergoing PCI are recorded.^19^ It covers >90% of the PCI procedures performed in Japan.^4^^,^^20^ The National Clinical Database, a Japanese nationwide prospective internet-based registry, incorporated the J-PCI registry in 2013. Each institution has a data manager responsible for collecting and entering data into an online database system. Data registration in the J-PCI registry is mandatory for board certification and renewal by CVIT, ensuring compliance with facility standards for the use of interventional devices. To ensure data robustness, CVIT conducts random audits (20 institutions annually) to evaluate the quality of registered data. A third-party central ethics committee (Clinical Research Promotion Network) approved the study protocol for the J-PCI registry. The present study was conducted in accordance with the Declaration of Helsinki, and the requirement to obtain written informed consent was waived because of the complete data anonymization and observational nature of this study.

### Benchmarking system

CVIT launched a simple, web-based benchmarking system in January 2018 to provide QIs in PCI at the institutional and national levels.^21^ The process whereby institutions tracked their QIs was centrally monitored within the national database system (National Clinical Database). The following 7 items were the QIs endorsed by the society to standardize the quality of PCI procedures in Japan^10^^,^^21^: 1) proportion of ACS; 2) proportion of nonelective PCI; 3) D2B time for STEMI; 4) pre-PCI antiplatelet agent use; 5) proportion of TRI; 6) pre-PCI stress testing performed in elective cases; and 7) proportion of side-branch PCI in elective procedures.

Given the apparent survival benefit of PCI in ACS and the predominant use of PCI in elective cases,^22^ the proportion of ACS and nonelective PCI is considered a quality metric in Japan. D2B time is a pragmatic target in patients with STEMI that has been advocated by academic societies.^23^^,^^24^ The importance of administering antiplatelet drugs before PCI is widely recognized; thus, antiplatelet therapy at the time of PCI is one of the QIs.^25^ Several randomized clinical trials have reported that TRI is associated with lower all-cause mortality and major bleeding compared with transfemoral intervention,^26^ suggesting that the transradial approach should be the default strategy in PCI, if possible. Previous studies have shown a unique practice pattern in Japan that noninvasive stress testing before PCI is infrequently performed,^27^ and its low use may result in a high likelihood of “rarely appropriate” PCI.^28^ Similarly, from the standpoint of the appropriate use criteria, elective PCI procedures only for side branches, defined as the American Heart Association classification of coronary artery segments 4AV, 4PD, 9, 10, 12, 13, 14, and 15,^29^ are unlikely to be “appropriate.”^28^ Therefore, the performance of pre-PCI stress testing and intervention for side branches are a quality metric. Fractional flow reserve measurement was not included as pre-PCI stress testing. Since January 2019, site-level data managers have been encouraged to review the benchmarking system online to recognize QIs at their institutions compared with the national average and to comment on their institutional metrics. In the present study, a benchmarking system review was defined when the manager at an individual center logged in to the dedicated web site (Supplemental Figure 1). Comments from local centers in reaction to the institutional statistics were provided narratively.

### Study population

Between January 2019 and December 2021, 734,379 PCI procedures at 1,194 institutions or departments were registered in the J-PCI registry. Patients underwent PCI for ACS and in elective cases. Patients aged <20 or ≥100 years and those with missing data on in-hospital mortality (n = 115) were excluded. Accordingly, 734,264 patients were included in this study. When a patient underwent PCI at a center at which the benchmarking system was checked, the patient was considered to belong to the benchmarking system review group. Patients were grouped according to the implementation of the benchmarking system review in 2019 and 2020 and during the 3-year study period. The definitions of the variables in the J-PCI registry are available online.^19^ In general, intracoronary imaging and contemporary-generation drug-eluting stents are predominantly used for PCI in current Japanese clinical practice.^30^, ^31^, ^32^, ^33^, ^34^ Academic hospitals are defined as university hospitals and affiliated institutions.

### Outcomes and statistical analysis

A schematic diagram of the study flow is shown in the Central Illustration. The primary aim of this study was to determine whether a benchmarking system review is associated with changes in QIs. To characterize the changes in QIs, patients undergoing PCI at institutions at which the benchmarking system was reviewed in 2019 were grouped into the benchmarking system review group, and the associations between the presence or absence of a benchmarking system review in 2019 and changes in the 7 QIs from 2019 to 2020 were descriptively compared at the patient level. In addition, to characterize institutions at which the benchmarking system was likely to be reviewed, patients undergoing PCI at centers at which the benchmarking system was reviewed during the 3-year study period were categorized into the benchmarking system review group, and hospital, patient, lesion, and procedural characteristics were compared between the review and no-review groups.

**Central Illustration:**
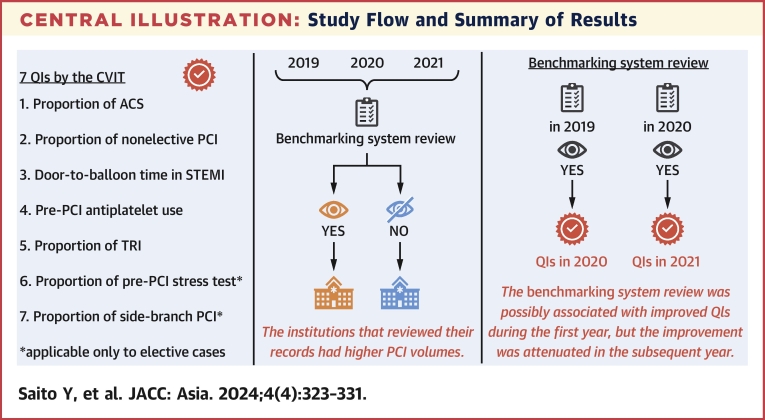
Study Flow and Summary of Results The Japanese Association of Cardiovascular Intervention and Therapeutics (CVIT) launched a simple, web-based benchmarking system in 2018 to provide 7 quality indicators (QIs) for percutaneous coronary intervention (PCI) at the institutional and national levels. The likelihood of benchmarking system review at the hospital level was associated with institutional PCI volumes. Benchmarking system review was possibly associated with the improvement of QIs during the first year, although the potential effect was attenuated in the subsequent year. ACS = acute coronary syndrome; STEMI = ST-segment elevation myocardial infarction; TRI = transradial intervention.

Statistical analyses were performed using R version 4.0.5 (R Foundation for Statistical Computing). Continuous variables are expressed as the mean ± SD when normally distributed and as median (Q1-Q3) when not normally distributed. Normality of distribution was visually evaluated using histograms. Categorical data are expressed as frequency (percentage). Continuous variables were compared using Student’s *t*-test or the Wilcoxon rank sum test, and categorical data were evaluated using the chi-square or Fisher exact test. The Fisher exact test was used when cell counts were <5. Clinical presentations including elective PCI, ACS, STEMI, non-STEMI, and unstable angina were compared between the review and no-review groups as individual variables. Statistical significance was set at *P* < 0.05.

## Results

Between 2019 and 2021, the benchmarking system was reviewed at least once AT 742 of 1,194 institutions (62.1%). The median number of benchmarking system reviews during the 3-year study period was 4 (Q1-Q3: 2-7), ranging from 0 to 56. Of the 734,264 included patients, 591,127 (80.5%) underwent PCI at the centers at which the benchmarking system was reviewed from 2019 to 2021. The institutions that reviewed the benchmarking system had higher PCI volumes than their counterparts (Table 1). Tables 2 and 3 list the patients, lesions, and procedural characteristics. The proportion of ACS was lower, and cardiogenic shock and cardiac arrest were more frequent at institutions at which the benchmarking system was reviewed than at their counterparts (Table 2). Although some variables had statistically significant differences, lesion and procedural characteristics did not differ considerably between the 2 groups, although PCI procedures for left main coronary artery disease were more common at the institutions at which the benchmarking system was reviewed (Table 3). In the benchmarking system review group, the D2B time for STEMI was shorter, the proportion of TRI was lower, and the rate of pre-PCI stress testing in elective cases was higher than at their counterparts (Table 4). Examples of narrative comments from local institutions are shown in Supplemental Table 1.

**Table 1:** Hospital Characteristics

	Benchmarking System Review		*P* Value
	No	Yes	
Number of hospitals	452	742	
Number of academic hospitals	22 (4.9)	108 (14.6)	<0.001
Number of PCIs for ACS per year	29 (11-58)	97 (58-139)	<0.001
Number of elective PCIs per year	39 (13-74)	134 (78-205)	<0.001
Number of total PCIs per year	72 (27-138)	221 (136-335)	<0.001

Values are n, n (%), or median (Q1-Q3). Academic hospitals are defined as university hospitals and affiliated institutions.

ACS = acute coronary syndrome; PCI = percutaneous coronary intervention.

**Table 2:** Patient Characteristics

	All (N = 734,264)	Benchmarking System Review		*P* Value
		No (n = 143,137)	Yes (n = 591,127)	
Age, y	71.2 ± 11.3	71.3 ± 11.3	71.1 ± 11.3	<0.001
Women	172,380 (23.5)	34,419 (24.0)	137,961 (23.3)	<0.001
Hypertension	555,173 (75.6)	107,231 (74.9)	447,942 (75.8)	<0.001
Diabetes	330,710 (45.0)	64,229 (44.9)	266,481 (45.1)	0.157
Dyslipidemia	491,454 (66.9)	94,136 (65.8)	397,318 (67.2)	<0.001
Current smoking	220,284 (30.0)	43,913 (30.7)	176,371 (29.8)	<0.001
Chronic kidney disease	169,763 (23.1)	30,374 (21.2)	139,389 (23.6)	<0.001
Hemodialysis	52,634 (7.2)	9,147 (6.4)	43,487 (7.4)	<0.001
COPD	21,711 (3.0)	4,559 (3.2)	17,152 (2.9)	<0.001
Peripheral artery disease	58,549 (8.0)	9,914 (6.9)	48,635 (8.2)	<0.001
Previous heart failure	114,244 (15.6)	22,442 (15.7)	91,802 (15.5)	0.168
Previous PCI	327,009 (44.5)	62,013 (43.3)	264,996 (44.8)	<0.001
Previous CABG	23,714 (3.2)	3,848 (2.7)	19,866 (3.4)	<0.001
Clinical presentation				
Elective PCI	518,161 (70.6)	100,732 (70.4)	417,429 (70.6)	0.067
ACS	286,269 (39.3)	58,509 (41.3)	227,760 (38.8)	<0.001
STEMI	132,372 (18.2)	26,646 (18.8)	105,726 (18.0)	<0.001
NSTEMI	46,982 (6.5)	8,484 (6.0)	38,498 (6.6)	<0.001
Unstable angina	100,285 (13.8)	22,221 (15.7)	78,064 (13.3)	<0.001
ADHF	31,899 (4.3)	6,454 (4.5)	25,445 (4.3)	0.001
Cardiogenic shock	26,347 (3.7)	5,053 (3.5)	21,294 (3.7)	0.019
Cardiac arrest	14,854 (2.1)	2,600 (1.8)	12,254 (2.1)	<0.001

Values are mean ± SD or n (%). ACS includes unknown myocardial infarction in addition to STEMI, NSTEMI, and unstable angina.

ADHF = acute decompensated heart failure; CABG = coronary artery bypass grafting; COPD = chronic obstructive pulmonary disease; NSTEMI = non–ST-segment elevation myocardial infarction; STEMI = ST-segment elevation myocardial infarction; other abbreviations as in Table 1.

**Table 3:** Lesion and Procedural Characteristics

	All (N = 734,264)	Benchmarking System Review		*P* Value
		No (n = 143,137)	Yes (n = 591,127)	
Lesion characteristics				<0.001
1	470,625 (64.1)	91,509 (63.9)	379,116 (64.1)	
2	178,560 (24.3)	35,213 (24.6)	143,347 (24.3)	
3	82,621 (11.3)	16,262 (11.4)	66,359 (11.2)	
Left main trunk	28,787 (3.9)	4,646 (3.2)	24,141 (4.1)	
Lesion location				<0.001
RCA	242,118 (33.0)	46,950 (32.8)	195,168 (33.0)	
LAD/left main coronary artery	394,055 (53.7)	77,827 (54.4)	316,228 (53.5)	
LCx	177,568 (24.2)	34,304 (24.0)	143,264 (24.2)	
Bypass graft	2,866 (0.4)	363 (0.3)	2,503 (0.4)	
Year PCI performed				<0.001
2019	253,165 (34.5)	46,464 (32.5)	206,701 (35.0)	
2020	239,440 (32.6)	46,942 (32.8)	192,498 (32.6)	
2021	241,618 (32.9)	49,731 (34.7)	191,887 (32.5)	
Pre-PCI FFR	55,103 (7.5)	10,578 (7.4)	44,525 (7.5)	0.068

Values are n (%).

FFR = fractional flow reserve; LAD = left anterior descending coronary artery; LCx = left circumflex coronary artery; PCI = percutaneous coronary intervention; RCA = right coronary artery.

**Table 4:** Quality Indicators and In-Hospital Mortality

	Benchmarking System Review		*P* Value
	No (n = 143,137)	Yes (n = 591,127)	
Proportion of ACS	41.3	38.8	<0.001
Proportion of nonelective PCI	29.6	29.4	<0.001
D2B time for STEMI, min	75 (57-97)	71 (54-91)	<0.001
Antiplatelet therapy use	99.8	99.7	<0.001
Proportion of TRI	76.9	74.0	<0.001
Pre-PCI stress testing^a^	16.7	19.0	<0.001
Proportion of side-branch PCI^a^	21.0	21.1	0.395
In-hospital mortality	1.9	1.8	0.007

Values are % or median (Q1-Q3).

D2B = door-to-balloon; other abbreviations as in Tables 1 and 2.

a Applicable only to elective PCI cases.

In 2019, 335 institutions (28.1%) reviewed the benchmarking system. The changes in institutional QIs from 2019 to 2020 on the basis of whether the benchmarking system review was performed in 2019 are shown in Table 5. In the benchmarking system review group, the proportion of TRI increased by 2.3% from 2019 to 2020, whereas it increased by 0.7% in the group without benchmarking system review (Table 5). Similarly, the proportion of ACS and nonelective PCI procedures increased by 2.1% from 2019 to 2020 in the benchmarking system review group and 0.5% in their counterparts (Table 5). No apparent effect of the benchmarking system review in 2019 was observed on D2B time, antiplatelet use, pre-PCI stress testing, and the proportion of side-branch PCI in 2020 (Table 5). However, the effect of the benchmarking system review may have been attenuated in the following year. The benchmarking system was reviewed by 528 institutions (44.2%) in 2020. The proportion of TRI increased by 1.4% and 0.8% from 2020 to 2021 at centers with and without benchmarking system review, respectively, in 2020 (Table 6). D2B time in the STEMI group increased by a median of 1 minute in the group without benchmarking system review, whereas it increased by a median of 3 minutes in the benchmarking system review group (Table 6). Overall results are summarized in the Central Illustration.

**Table 5:** Benchmarking System Review in 2019 and Changes in Quality Indicators in a Following Year

	Benchmarking System Review in 2019			
	No		Yes	
	2019 (n = 162,171)	2020 (n = 156,282)	2019 (n = 90,994)	2020 (n = 83,158)
Proportion of ACS	38.7	39.2	38.1	40.2
Proportion of nonelective PCI	38.7	39.2	38.1	40.2
D2B time for STEMI, min	71 (54-91)	72 (55-92)	69 (53-89)	70 (53-90)
Antiplatelet therapy use	99.6	99.6	99.7	99.8
Proportion of TRI	73.7	74.4	72.8	75.1
Pre-PCI stress testing^a^	19.2	18.5	20.2	19.2
Proportion of side-branch PCI^a^	21.4	21.0	21.1	21.0
In-hospital mortality	1.6	1.9	1.9	2.1

Values are % or median (Q1-Q3).

Abbreviations as in Tables 1, 2, and 4.

a Applicable only to elective PCI cases.

**Table 6:** Benchmarking System Review in 2020 and Changes in Quality Indicators in a Following Year

	Benchmarking System Review in 2020			
	No		Yes	
	2020 (n = 84,325)	2021 (n = 86,991)	2020 (n = 155,115)	2021 (n = 154,627)
Proportion of ACS	41.1	41.3	38.8	39.2
Proportion of nonelective PCI	41.1	41.3	38.8	39.2
D2B time for STEMI, min	73 (55-92)	74 (57-96)	70 (53-90)	73 (57-96)
Antiplatelet therapy use	99.8	99.8	99.6	99.7
Proportion of TRI	76.1	76.9	73.8	75.2
Pre-PCI stress testing^a^	17.7	15.9	19.3	17.8
Proportion of side-branch PCI^a^	21.6	21.3	20.7	20.8
In-hospital mortality	2.1	2.1	1.9	1.8

Values are % or median (Q1-Q3).

Abbreviations as in Tables 1, 2, and 4.

a Applicable only to elective PCI cases.

## Discussion

The present data from a nationwide registry in Japan show that a web-based benchmarking system provided by CVIT, a professional society responsible for certifying both interventionalists and institutions, was reviewed by more than 60% of institutions across the country during the 3-year period immediately after the introduction of the QI benchmarking mechanism. Among the institutions at which the benchmarking system was reviewed, the PCI volume was higher, the proportion of ACS was lower, and cardiogenic shock and cardiac arrest were more frequent than at their counterparts. Among the 7 QIs, D2B time for STEMI was shorter, the proportion of TRI was lower, and the rate of pre-PCI stress testing in elective cases was higher in the benchmarking system review group than in the no-review group. The benchmarking system review was possibly associated with an improvement in QIs in the following year, particularly in the proportion of TRI, whereas the potential effect was attenuated in the subsequent year. The present study results suggest the feasibility of a simple, web-based, academia-led benchmarking system and, at the same time, room for improvement in QIs in PCI.

### QIs in PCI

QIs have been widely used in the field of PCI for standardized dissemination and improvement of care quality. For instance, as a readily assessable measure and pragmatic target, D2B time has been advocated in international guidelines.^23^^,^^35^ Given its prognostic impact,^36^ stakeholders, including academic societies and the public, have moved forward with the widespread adoption of D2B time (≤90 minutes) as a QI for STEMI, leading to a significant decline in D2B time at the national level.^37^, ^38^, ^39^ Although adherence to QIs is associated with better clinical outcomes in the setting of ACS,^3^^,^^11^, ^12^, ^13^, ^14^ feasible and efficient QIs in ACS and PCI remain uncertain. The European Society of Cardiology updated QIs for acute myocardial infarction in 2020, including center organization, reperfusion and invasive strategy, in-hospital risk assessment, antithrombotic treatment, secondary prevention, patient satisfaction, and composite indicators and mortality.^1^ In the United States, academic societies proposed 11 QIs as PCI-related process measures at the hospital level, such as D2B time, antiplatelet agent use, and others.^40^ Similarly in Japan, CVIT proposed 7 QIs in PCI, such as proportion of ACS and TRI and D2B time for STEMI, and introduced a simple, web-based benchmarking system in 2018.^10^^,^^21^ In the present study, therefore, we evaluated the impact of the benchmarking system on QIs.

### Benchmarking and feedback systems on QIs

To improve the quality of care in ACS and PCI, numerous interventional approaches, including information communication technology for health, training medical staff members, site performance assessment and feedback, financial incentives, and patient education, have been previously tested.^16^^,^^17^ However, there are substantial variations in the types, effectiveness, and implementation of QI strategies for patients with cardiovascular diseases.^15^ Although several randomized studies have shown a significant effect of multifaceted intervention on the improvement in QIs,^24^^,^^41^ a meta-analysis indicated that the clinical benefit of interventions for quality improvement may be modest in patients with ACS.^16^ Among the interventional approaches, data feedback is a traditional and common pathway to achieve improvements in adherence to QIs and subsequent outcomes. In the nonrandomized FITT-STEMI (Feedback Intervention and Treatment Times in STEMI) study, regular interactive feedback sessions with local STEMI management teams were associated with better care quality during the 10-year study period, for instance, in contact-to-balloon time <90 minutes at baseline and at follow-up (37.2% vs 53.7%; *P* < 0.001).^42^ In addition, a previous randomized control trial demonstrated that the use of achievable benchmarks for physician feedback improved QIs.^43^ In contrast, the randomized AFFECT (Administrative Data Feedback for Effective Cardiac Treatment) trial in Quebec, Canada, showed that one-time feedback provided as a report card containing information on 12 QIs at each hospital compared with those at province average and sent to a site manager was not effective for quality improvement regarding care of patients with acute myocardial infarction.^44^ These findings suggest that feedback intervention is not uniform and that the effectiveness should be validated in individual approaches. The benchmarking system provided by CVIT, in which only 7 QIs can be reviewed online by a site manager, is apparently similar to and simpler than that used in the AFFECT trial. In the present study, the web site of the benchmarking system was reviewed by more than 60% of the institutions during the 3-year period, indicating the feasibility of the system and room for further improvement in recognition and dissemination. The institutions at which the benchmarking system was reviewed in this study were likely to be academic hospitals and to have high PCI volumes. It is well known that at small-volume hospitals, care quality and clinical outcomes vary widely,^5^^,^^6^ possibly because of lower compliance with data feedback.

In the present study, benchmarking system review was potentially associated with improvements in QIs, particularly in the proportion of TRI. Although Japan already had a high rate of TRI compared with Western countries, the benchmarking system review possibly contributed to further incremental improvements. Meanwhile, it may be logistically challenging to increase the proportion of ACS and nonelective PCI by checking the benchmarking system. Given this extremely high percentage, there may be no room for improvement with antiplatelet therapy. D2B time for STEMI is one of the most common QIs in PCI; thus, an improvement in D2B time by the benchmarking system review was expected but was not observed. From 2019 to 2020, D2B time was slightly increased irrespective of the benchmarking system review, which may be explained by the COVID-19 pandemic. More intensive approaches may be needed to shorten D2B for STEMI.^24^ Although the proportion of TRI increased, the absolute change in percentage was relatively small (2.3% from 2019 to 2020 and 1.4% from 2020 to 2021 in the feedback system review group). In our international comparative study using J-PCI and U.S. registry data, we observed that the percentage of individual QIs remained relatively stable between 2013 and 2016.^10^ Thus, although the absolute numbers of changes appear small, we believe that these postfeedback alterations have clinical significance. Of note, the effect of the benchmarking system review on the proportion of TRI was apparently attenuated from the early to the late period of the present study, potentially confirming the fact that the effect of feedback intervention was null.^42^^,^^45^ In the aforementioned FITT-STEMI study, the effect of interactive feedback was more evident in the early period of study participation.^42^ Therefore, further efforts are warranted to develop sustainable systems to improve care quality in PCI.

### Study limitations

Despite the large sample size, this was an observational, nonrandomized study. Benchmark system reviews have many confounding factors. Thus, a cause-effect relationship cannot be established. In this study, we used patient-level rather than site-level analysis to evaluate the benchmarking system review. As the web-based benchmarking system is domestically and uniquely developed, our results may not be directly extrapolated to clinical practice in other regions. Although the present study indicated the potential of the benchmarking system to improve some QIs at the hospital level, it is still unestablished whether the improvement in QIs translates into enhanced clinical outcomes. A previous study demonstrated that annual PCI-related mortality rates at the hospital level may not reliably reflect hospital quality or predict practice changes or patient hospital selection.^46^ Interestingly, hospitals with high or low PCI-related mortality rates tended to regress to the mean the following year, indicating a lack of consistent association between a hospital’s risk-adjusted mortality rate and its future performance. To assess the practical effect of the benchmarking system on clinical outcomes would likely require long-term data collection and analysis, which extends beyond the scope of our present study design. Additionally, the first patient with COVID-19 was reported in January 2020, and the pandemic occurred thereafter in Japan,^20^ which may have affected the results of the present study. The impact of COVID-19 on daily practice of PCI in Japan was investigated using data from the J-PCI registry, showing that daily case volume of PCI decreased by 6.7% in 2020 compared with 2019; more severe clinical presentations of PCI patients were observed in 2020, such as STEMI, acute heart failure, cardiogenic shock, and cardiac arrest; and adjusted in-hospital mortality was increased in patients treated in 2020 compared with those treated in 2019.^20^ Consistent with these findings, our present study also revealed delays in D2B time during the COVID-19 pandemic. However, the majority of QI parameters related to elective PCI cases remained largely consistent.

## Conclusions

The benchmarking system led by an academic society in Japan was likely to be reviewed at academic hospitals and centers with high PCI volumes. The implementation of a benchmarking system review and the use of a simple web-based platform demonstrated the potential to improve QIs in the subsequent year, particularly in the proportion of TRI. However, this association may have been attenuated in subsequent years. Therefore, dedicated efforts should be made to develop effective and sustainable interventions to enhance the quality of PCI care.

Perspectives **COMPETENCY IN MEDICAL KNOWLEDGE:** The present study showed the feasibility of simple, web-based, academia-led benchmarking system in QIs in PCI in Japan. Centers with higher PCI volumes were likely to review the benchmarking system.

**TRANSLATIONAL OUTLOOK:** The benchmarking system review was possibly associated with improvement in adherence to QIs. Dedicated efforts should be made to develop effective and sustainable interventions for enhancing care quality in PCI.

## Funding Support and Author Disclosures

This study was supported by a grant from the Chiba Foundation for Health Promotion and Disease Prevention and the Health and Labor Sciences Research Grants (21FA1015). Dr Inohara is an employee of Eli Lilly Japan. Dr Kohsaka has received investigator-initiated grants from Novartis and AstraZeneca; and has received lecture fees from Bristol Myers Squibb. Dr Kumamaru has received consultation fee from EPS Corporation; has received speaker fee from Chugai Pharmaceutical; and is affiliated with the Department of Health Quality Assessment at the University of Tokyo, a social collaboration department supported by the National Clinical Database, Johnson & Johnson, Nipro Corporation, and Intuitive Surgical Sàrl. Dr Ishii has received lecture fees from AstraZeneca, Bayer Pharmaceutical, Boehringer Ingelheim Japan, Bristol Myers Squibb, Daiichi-Sankyo, Merck Sharpe & Dohme, Mitsubishi Tanabe Pharma, Mochida Pharmaceutical, Novartis Japan, and Pfizer Japan. Dr Amano has received lecture fees from Astellas Pharma, AstraZeneca, Bayer, Bristol Myers Squibb, and Daiichi-Sankyo. All other authors have reported that they have no relationships relevant to the contents of this paper to disclose.
