# Supplementary material for: Benchmarking System Monitoring on Quality Improvement in Percutaneous Coronary Intervention: A Nationwide Registry in Japan
Source: JACC Asia. 2024 Feb 20;4(4):323–31. doi: 10.1016/j.jacasi.2023.12.003 (PMC11035937; doi:10.1016/j.jacasi.2023.12.003)
Supplement: Supplemental Figure 1 and Supplemental Table 1 [file mmc1.docx]

**Supplemental Figure 1. Website of the online benchmarking system endorsed by the NCD and CVIT**

**
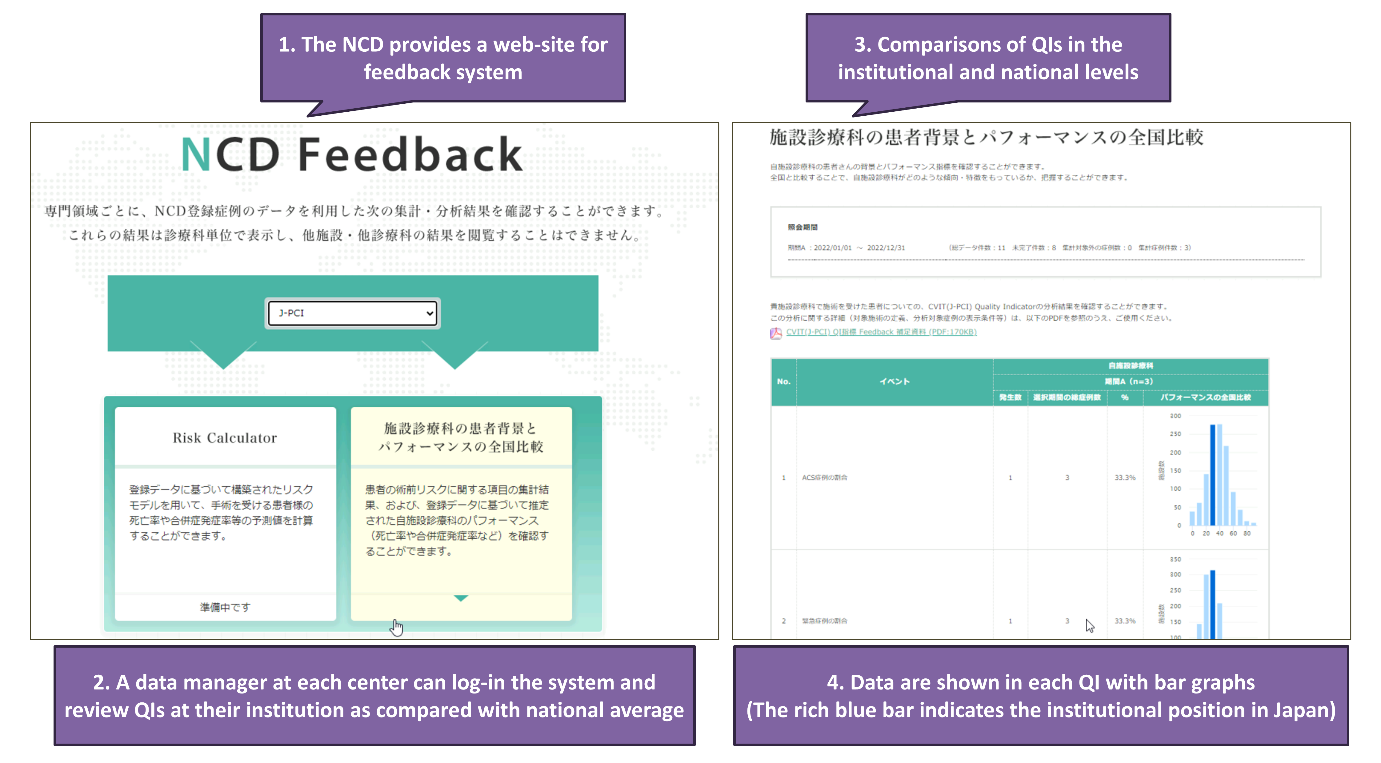
**

CVIT = Japanese Association of Cardiovascular Intervention and Therapeutics; NCD = National Clinical Data.

**Supplemental Table 1.** **Exemplary Narrative Comments from J-PCI Institutions Following Review of Individual Benchmarking Data**

| Although the rate of transfemoral PCI in our institution exceeded the national standard, a substantial proportion of patients with STEMI achieved D2B time ≤90 min. This achievement may be attributed to effective collaborations between doctors and medical staff, and an established on-call system. Notably, the rates of preoperative antiplatelet drug administration and ischemia evaluations in elective cases surpassed the national averages. We believe that the proactive use of non-invasive and invasive ischemia testing can aid in determining appropriate indications of PCI. We remain committed to utilizing the NCD feedback system as a valuable reference when formulating treatment strategies. |
| --- |
| The rate of emergency PCIs is relatively low (21.6%) in our institution and thus, we aim to strengthen the emergency department, catheterization staff, and on-call systems. We understand that the rate of PCI in the branches rather than in the major coronary arteries in elective cases was relatively high (22.1%), and ischemia evaluation in elective cases was infrequently performed (55.8%). We intend to improve these statistics in the future. |
| We were concerned that our hospital had a lower rate of radial artery access compared with the national average. This might result in a recent increase in the mortality rate in patients with acute myocardial infarction in our institution. We plan to increase radial artery access shortly. |
| Although statistics in our hospital do not significantly deviate from the national average overall, the rate of ischemia evaluation in elective cases was somewhat low. In the future, we will be more proactive in evaluating lesions suitable for PCI, using FFR and non-invasive stress testing. There is room for improvement regarding D2B time in STEMI, and we would like to improve this by cooperating more closely with the emergency department. |
| Although the proportion of patients with ACS was low in our institution, many hospitals in Sapporo City are available for such patients, probably resulting in the low ACS rate. The rate of the radial artery approach was also low, because femoral artery approach is commonly used in patients on hemodialysis and in an emergency setting in our institution. |
| This year, D2B time ≤90 min accounted for 51.5% of patients with STEMI in our institution, which was substantially decreased from that in the previous year, probably due to the COVID-19 pandemic. Screening examinations with antigen and PCR tests as well as chest CT evaluation were required before the catheterization, particularly in patients with relatively stable vital signs. The situation remains unchanged today. |
| The rate of ischemia evaluation in patients undergoing elective procedures was impressively high (97%) in our institution, and we believe that the assessment was appropriate. Our goal in the near future is to consistently provide appropriate PCI to patients who require it, while actively avoiding unnecessary procedures. |
| The high in-hospital mortality in patients with acute myocardial infarction may be attributed to the characteristics of our hospital where many elderly patients and those with poor health status are selectively transported. |
| We aim to improve the low proportion of D2B time ≤90 min in patients with STEMI. However, this low rate was largely due to a system delay associated with the COVID-19 pandemic. Our institution prioritized screening tests and infection prevention before PCI. |
| PCIs for patients with ACS are frequent in our hospital, particularly in those with cardiogenic shock and cardiac arrest. Thus, many patients with STEMI did not achieve D2B time ≤90 min. Nevertheless, we recognize the need for improvement in this regard. Although our historical rate of preoperative antiplatelet drug prescription has been low, this has improved to 78%. The proportion of ischemia evaluation in elective cases is currently low at 56%. Therefore, we now discuss indications of elective PCIs at a conference preoperatively. |
| To improve D2B time in our institution, our plan is to share information with our staff on an increasing trend of D2B time. We also aim to actively utilize FFR/NHPRs and non-invasive ischemia testing to increase the proportion of ischemia evaluation in elective cases for at least meeting the national average. |
| In our institution, a significant portion of patients on hemodialysis undergo PCI procedures via the femoral artery, leading to a lower rate of radial artery approach as compared with the national average. Additionally, the use of directional coronary atherectomy and rotational atherectomy with a large burr usually requires 8 Fr catheters through the femoral artery. The rate of ACS was relatively low in our institution, and D2B time was longer than the national average. Thus, efforts are ongoing to establish a better system that allows cardiologists to actively engage in medical care at an earlier stage in a setting of ACS. |
| The low rate of radial artery access may be attributed to the frequent use of femoral artery approach, particularly in emergency PCIs. The low rate of ischemia evaluation in elective cases may have to be improved. We believe that the proportion of D2B time ≤90 min in our institution is sufficiently high as compared to the national average. |
| We preoperatively conduct comprehensive examinations, including FFR and non-invasive ischemia testing, before PCI in elective cases and prioritize less invasive procedures with the radial artery approach being our preferred choice. In 2018, we established a local electrocardiogram transmission system during medical transportation before hospital arrival. We believe that the initiative significantly contributed to the reduction in D2B time in our hospital in patients with STEMI. |
| We plan to enhance collaboration between physicians in the cardiology and emergency departments to increase the proportion of STEMI patients with D2B time ≤90 min. |
| D2B time in patients with STEMI has been prolonged due to logistic challenges in our institution. We aim to establish a cooperative system between primary care physicians and cardiologists and to promote staff education. The rate of ischemia evaluation in elective procedures in our hospital surpassed the national average, and we are confident in terms of the appropriateness of PCI procedures. |
| The NCD feedback system indicated a high rate of ischemia evaluation in our institution in elective cases. We perform PCI in patients with stable coronary artery disease only when myocardial ischemia is documented. Despite the nationwide goal of achieving D2B time ≤90 min in STEMI, our efforts currently aim at further reducing the metric. |

ACS = acute coronary syndrome; COVID-19 = coronavirus disease 2019; D2B = door-to-balloon; FFR, fractional flow reserve; NCD = National Clinical Data; NHPR = non-hyperemic pressure ratio; PCI = percutaneous coronary intervention; PCR = polymerase chain reaction; STEMI = ST-elevation myocardial infarction.
